# Chitin Triggers Tissue-Specific Immunity in Wheat Associated With Fusarium Head Blight

**DOI:** 10.3389/fpls.2022.832502

**Published:** 2022-02-09

**Authors:** Guixia Hao, Helene Tiley, Susan McCormick

**Affiliations:** USDA, Agricultural Research Service, National Center for Agricultural Utilization Research, Mycotoxin Prevention and Applied Microbiology Research Unit, N. University St., Peoria, IL, United States

**Keywords:** Fusarium head blight, *Fusarium graminearum*, chitin, reactive oxygen species, plant defense responses

## Abstract

*Fusarium graminearum* is one of the primary causal agents of Fusarium head blight (FHB) on wheat and barley. FHB reduces grain yield and contaminates grain with various mycotoxins, including deoxynivalenol (DON). DON acts as a virulence factor to promote the fungus passing the rachis node and spreading throughout the head of wheat but not barley. Reactive oxygen species (ROS) are one of the earliest defense responses during plant and pathogen interactions. However, the complex roles of ROS during FHB development remain unclear. We investigated immune responses in wheat triggered by chitin, a major component of fungal cell walls. Although no ROS burst was detected in chitin-treated wheat leaves from eight tested varieties, a robust ROS peak was triggered by chitin in tested barley leaves. Interestingly, ROS were induced by chitin in wheat rachises and rachis nodes, which are critical barriers for FHB spread in wheat. We demonstrated that ROS were induced in wheat rachis nodes from both FHB susceptible and resistant wheat varieties. Further, we showed different defense gene expression patterns in rachis nodes and wheat heads treated with chitin, and wheat heads inoculated with *F. graminearum*. Our study showed the tissue-specific immune responses induced by chitin in wheat, which may play an important role during *F. graminearum* infection.

## Introduction

Plants have evolved multi-layered immune responses toward microbial pathogen attacks. The perception of pathogen (microbe or damage)-associated molecular patterns (PAMPs, MAMPs, or DAMPs) by membrane-bound pattern recognition receptors (PRRs) leads to PAMPs−, MAMPs−, or DAMPs-triggered immunity (PTI, MTI, or DTI), including calcium ion (Ca^2+^) influx, reactive oxygen species (ROS) burst, and defense gene activation (Bigeard et al., 2015). Chitin is a long-chain polymer of *N*-acetylglucosamine, an amide derivative of glucose. It is a primary component of cell walls in fungi, the exoskeletons of arthropods, and the shells of crab and shrimp. Chitin is one of the best studied PAMPs to induce plant immunity against invading pathogens. Rapid ROS production in response to pathogen attack is critical to establish plant immune responses. In addition to the direct toxic effects on pathogens, ROS function as cellular signaling molecules to trigger plant defense responses, such as cell wall strengthening, hormone synthesis, and programmed cell death. ROS are oxygen-containing reactive radicals, such as superoxide anion (·O_2_^−^), hydrogen peroxide (H_2_O_2_), and hydroxyl radical (·OH) (Grant and Loake, 2000). In response to MAMPs treatment, activated cell wall peroxidases and plasma membrane NADPH oxidases, also known as Respiratory Burst Oxidase Homologs (RBOHs), catalyze and convert ·O_2_^−^ and ·OH to H_2_O_2_ in the extracellular space (Keller et al., 1998; Torres et al., 2002; Bienert and Chaumont, 2014). H_2_O_2_ is one of the most stable ROS in plants and can diffuse through cell membranes. The quantity of H_2_O_2_ has been measured in multiple plant species using leaf disks and cell cultures by luminol-based chemiluminescence in the presence of the oxidizing agent horseradish peroxidase (Trujillo, 2016; Bredow et al., 2019). Due to the critical role of ROS during plant and pathogen interactions, effectors secreted from many pathogens target multiple steps in the ROS signaling pathway to promote infection and disease (Jwa and Hwang, 2017).

Typically, plants produce a biphasic ROS induction following pathogen perception. The first phase is observed in both compatible and incompatible plant-microbe interactions. The stronger second phase is induced during an incompatible interaction, which has a crucial role in programmed cell death (Grant and Loake, 2000). In addition, the roles of ROS during plant-pathogen interactions depend on pathogen lifestyles and plant types. ROS have been shown to be a powerful weapon against biotrophic pathogen infection by inducing programmed cell death to limit pathogen spread. Conversely, during necrotrophic pathogen infection, ROS associated cell death may promote pathogen colonization and disease development. At low/moderate levels, ROS act as secondary messengers in intracellular signaling cascades that mediate plant defense responses. Many studies have shown that PAMPs-induced ROS can strengthen plant defense responses. Chitin treatments have been applied to various plants to enhance defense responses and reduce diseases (Faoro et al., 2008; El Hadrami et al., 2010; Kheiri et al., 2016). In addition, studies have demonstrated PTI was compromised in Arabidopsis (*Arabidopsis thaliana*) ROS mutants *Atrboh* (Torres et al., 2002).

Chitin-mediated immunity has been demonstrated between multiple fungal-plant interactions (Shinya et al., 2015). In Arabidopsis, the LysM domain receptor kinase (RK) CHITIN ELICITOR RECEPTOR-LIKE KINASE 1 (CERK1) is the PRR for chitin (Miya et al., 2007; Wan et al., 2008). In rice (*Oryza sativa*), both the LysM domain containing receptor-like protein CHITIN ELICITOR BINDING PROTEIN (CEBiP) and CERK1 are required for chitin recognition and to mediate resistance to fungal pathogens (Kaku et al., 2006; Shinya et al., 2012). Compared to the model plants Arabidopsis and rice, limited information is available in wheat about chitin-mediated signaling. Studies showed that CEBiP and CERK1, homologs of rice chitin receptors, play an important role in chitin recognition and fungal resistance in wheat (Lee et al., 2014). The expression of NADPH oxidase genes, wheat *TaRbohD* and *TaRbohF*, is induced upon rust fungus *Puccinia triticina* infection (Dmochowska-Boguta et al., 2013). Several PTI marker genes have been defined that are induced by PAMPs in wheat (Schoonbeek et al., 2015). For example, the syntaxin *TaROR2*, which shares homology with Arabidopsis *PEN1* and barley *HvROR2*, is involved in defense against pathogens (Collins et al., 2003). *TaMPK3* encoding a MAP kinase is involved in resistance to *Mycosphaerella graminicola* (Rudd et al., 2008). *TaPDR2* is also induced by *Magnaporthe* isolates during wheat infection (Tufan et al., 2009). The ubiquitin ligase genes *PUB23-like*, *CMPG1*-*like,* and *cupredoxin-like* were highly induced upon PAMP treatment (Kirsch et al., 2001; Trujillo et al., 2008; Schoonbeek et al., 2015).

*Fusarium graminearum* is considered a hemibiotrophic pathogen. During infection, *F. graminearum* produces deoxynivalenol (DON), which facilitates FHB spread throughout the wheat head (Jansen et al., 2005). Fungal mutants deficient in DON production cannot pass the rachis node to spread to the neighboring uninfected spikes (Proctor et al., 1995). In addition, infiltration of wheat leaves with DON induced ROS production, defense gene expression, and cell death (Desmond et al., 2008). During *F. graminearum* infection, ROS may function as a double-edged sword. Enhanced ROS burst can inhibit initial pathogen infection; however, strong ROS production at later stages may promote disease spread and toxin production. Prior studies have shown that *F. graminearum* increases DON production under oxidative stress environments (Ponts et al., 2006; Audenaert et al., 2010). In contrast, another study showed that the expression of *TRI5*-GFP was not induced with the addition of H_2_O_2_, suggesting H_2_O_2_ did not increase DON production (Ilgen et al., 2009). Our recent studies demonstrated that several *F. graminearum* effectors can suppress chitin-trigged ROS burst and Bax-induced cell death *via* transient expression in *N. benthamiana* leaves. Deletion mutants of effectors, such as Arb93B and FGSG_01831, significantly reduced FHB and DON contamination compared to the wild type (Hao et al., 2019, 2020). Therefore, it is important to investigate the role of ROS during *F. graminearum* infection of wheat.

Initially, our goal was to investigate whether we can employ ROS production from wheat seedlings to predict FHB severity and prime FHB resistance with chitin treatments. However, we could not detect ROS burst in all tested wheat leaves treated with chitin. Since *F. graminearum* primarily infects wheat heads and causes FHB, therefore, we assessed ROS production in different wheat head tissues. Further, we investigated chitin-induced ROS production in wheat rachis nodes from FHB susceptible and moderately resistant varieties and examined the relationship between FHB severity, DON content, and ROS level in different wheat varieties. Furthermore, we determined PTI marker gene expression in wheat heads and rachis nodes after chitin treatment and during *F. graminearum* infection.

## Materials and Methods

### Plant Cultivation

Four FHB susceptible wheat varieties: Norm, Wheaton, MN11492, and Ulen, four moderately resistant varieties: Alsen, MN-08173, Shelly, and Sabin (the selection was based on wheat type II resistance; Hay et al., 2022), and six barley varieties: Golden Promise, Voyager, Golf, Yuma510–510, Chevron, and Sy Sirish were used. Seeds were surface sterilized, and the germinated seeds were planted in 7-inch pots as described (Hao et al., 2019, 2020). Briefly, SunShine Mix (Sun Gro Horticulture, Agawam, MA) was used with the addition of 100 g Osmocote and 15 g Micromax in 5 L soil. The plants were grown in a controlled growth chamber at 20–23°C with a photoperiod of 16 h and 50% relative humidity. Plants were watered every day and fertilized every 2 weeks with Peter’s 20:20:20 (Grace-Sierra Horticultural Products, Milpitas, CA) for 6 weeks.

### ROS Measurement

ROS assays were performed as described (Hao et al., 2019). Briefly, wheat and barley leaves were removed from 7- to 12-day-old seedlings. Leaf stripes (3 × 3 mm) were sliced using a razorblade. Flowering heads were dissected into lemmas, paleae, rachises, and rachis nodes (Supplementary Figure S1). Three heads were used for each variety. Three to four spikelets were removed from the middle of the head. The rachis node was dissected by cutting on both sides of the junction between the rachis and spikelet. Lemma and palea were gently separated using tweezers. Using a razor blade, the awn of the lemma was removed, and the lemma was cut into four quadrants. The palea was also cut, either into two or four pieces depending on its size. Rachises were cut and used for assays. A total of 12 pieces (three technical replicates from one head) for each tissue were used on one plate. Each piece was placed in an individual well of a clear 96-well plate with 200 μl of water per well at room temperature overnight and covered with aluminon foil. The next day the water was removed, and ROS production was detected in a solution containing 100 μg/ml crab chitin, 20 mM L012, and 1 μg/ml of horseradish peroxidase (Sigma, St. Louise, CO). Leaves and head tissues without chitin treatment served as a negative control. Luminescence was measured over a period of 40–60 min using the Synergy HT and Gen5 software (BioTek Instruments Inc. Winooski, VT). The assays were repeated at least three times.

### Defense Marker Gene Expression in Wheat Heads and Rachis Nodes Treated With Chitin

To examine gene expression triggered by chitin, wheat heads on live plants were dipped into 100 μg/ml chitin (Sigma, St. Louise, CO) solution containing .02% Tween 20. Heads dipped in .02% Tween 20 served as controls. Three biological replicates were performed for each treatment. Treated and control heads were collected at 1, 3, 6, and 24 h for RNA isolation. Rachis nodes were dissected from flowering heads and placed in 1.5 ml Eppendorf tubes with 500 μl water and were left overnight to avoid gene expression in response to wounding as described (Schoonbeek et al., 2015). The following day, the water was replaced with fresh water (control) or 100 μg/ml crab chitin. Each treatment contained approximately 80 rachis nodes. Samples were collected at 0, 30, 60, and 120 min after treatment and ground immediately in liquid nitrogen for RNA isolation. RNA was isolated, cDNA was synthesized, and qPCR was performed as described (Hao et al., 2019). Briefly, RNA was extracted from pulverized wheat head tissue using Trizol combined with column purification and on-column digestion using an RNA purification kit (Thermo Fisher Scientific, Waltham, MA). First-strand cDNA was synthesized, and qPCR was conducted in a CFX96 Real-time PCR Detection system (Bio-Rad, Hercules, CA). Gene expression levels were calculated with the 2^−∆∆Ct^ values using cDNA from water or Tween 20 treated controls. The following genes were examined in our study: *TaCERK1*, *TaCEBiP*, *TaRbohD*, *TaRbohF*, *TaMPK3*, *TaWRKY23-like, TaPDR2*, *TaPUB23-like*, *TaCAMP1-like, TaROR2*, *Tacupredoxin-like,* and three hormone pathway genes: *TaPR1*, *TaPAL1,* and *TaLOX*1. Primers for these genes are listed in Supplementary Table S1. The wheat gene *TaGAPDH* was used as the endogenous control for normalization of gene expression.

### Defense Marker Gene Expression in Wheat Heads Infected by *F. graminearum*

A susceptible wheat variety, Norm, was used for *F. graminearum* inoculation and gene expression. The *F. graminearum* strain PH-1 was maintained on V8 agar plates. Conidia were prepared from 4-day-old mung bean culture and adjusted to the concentration of 1 × 10^5^ conidia/ml in 0.02% Tween 20 solution. Dip inoculation was performed as described (Hao et al., 2019). Three biological replicates were conducted, and heads were collected at 1, 3, 6, and 24 hpi. Tween 20 solution (.02%) treated heads were collected and served as controls. Heads were collected randomly at each time point, immediately frozen in liquid nitrogen, and stored at −80° prior to RNA isolation. The same set of genes tested during chitin treatment experiments was examined by RT-PCR as described above.

### FHB Pathogenesis Assays and DON Measurement

Seven wheat varieties, Norm, Wheaton, Ulen, Alsen, MN-08173, Shelly, and Sabin, were used for disease assays. *F. graminearum* PH-1 spores were prepared and adjusted to a concentration of 10^5^ conidia/ml. At anthesis, approximately BBCH 64–65 (Lancashire et al., 1991), a single floret from each head was inoculated with 10 μl of conidia solution. To maintain high humidity, the heads were covered in a plastic bag for 3 days after inoculation. At least 10 wheat heads were inoculated for each variety. Disease was scored by visualized FHB symptoms at 7-, 14-, and 21-days post-inoculation (dpi). Heads were collected at 21 dpi. One to two florets from individually inoculated heads were used for DON extraction. DON content was measured using GC/MS analysis (Hao et al., 2019). Experiments were repeated with similar results.

### Statistical Analysis

Statistical analysis was performed using JMP15. Means from biological replicates for ROS, disease, and gene expression were compared using one-way ANOVA, Dunnett’s method, and Tukey’s honestly significant difference *post-hoc* test. Using JMP 15, two-variable scatter plots were created. For each pair of variables, a Bivariate-Scatterplot and Regression analysis was performed. A linear fit was then applied to the model. The R-squared values were obtained from the summary of fit analysis, and the values of *p* were obtained as the Prob. > *F* in the ANOVA section of the linear fit analysis.

## Results

### No ROS Burst Detected in Chitin-Treated Wheat Leaves

**Figure 1:**
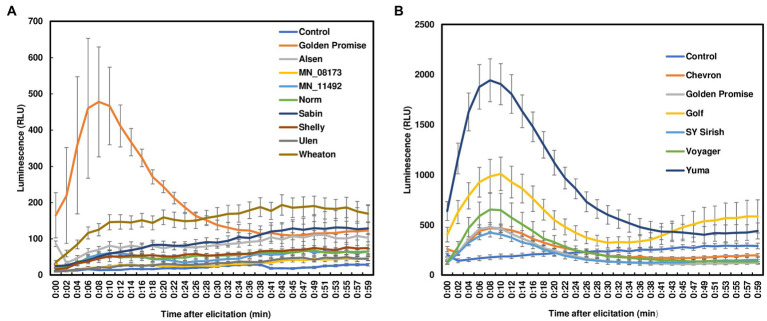
ROS burst in wheat and barley leaves with chitin treatment. **(A)** Four FHB moderately resistant (Alsen, Sabin, Shelly, and MN-08173) and four FHB susceptible (Norm, MN11492, Wheaton, and Ulen) wheat varieties were tested. Golden Promise served as a positive control; **(B)** Barley varieties (Golden Promise, Voyager, Golf, Yuma510–510, Chevron, and Sy Sirish) were used. Leaves were collected from 7- to 12-day-old plants. Approximately 3 × 3 mm leaf pieces were sliced and treated with crab chitin (100 μg/ml). ROS were monitored using a chemiluminescence assay with L012 as a substrate. The plates were run on a 96-well plate reader and signals (RLU, relative light unit) were recorded for about 60 min. The data represent means ± standard error (*n* = 12). The experiments were repeated at least three times with similar results.

ROS burst, one of the earliest PTI responses, exhibits a rapid and transient accumulation after perception of pathogen attack. ROS can be induced by pathogen invasion or *via* elicitor treatments. Fungal chitin is one of the best studied elicitors during plant defense signaling. To assess ROS production in wheat leaves, we treated leaf sections from 7- to 12-day-old seedlings using crab chitin (100 μg/ml). No ROS burst was observed in wheat leaves from four FHB moderately resistant or four susceptible varieties with chitin treatment (Figure 1A). It is worth noting that resistance to *F. graminearum* infection has been classified into two types. Resistance to initial infection is considered type I resistance. Resistance to FHB spread is classified as type II resistance. These varieties were selected based on type II resistance because wheat lacks type I resistance. To further determine whether the lack of ROS detection in wheat leaves was due to low chitin sensitivity, different chitin concentrations (100 μg/ml to 2,000 μg/ml) were used to treat Norm leaves. However, no ROS burst was detected in wheat leaves regardless of the chitin concentration applied (Supplementary Figure S2). For comparison, we also treated barley leaves from six varieties. All barley varieties have strong type II resistance but lack type I resistance. In contrast to wheat, a typical ROS burst was induced in all chitin-treated barley leaves. ROS peaks were observed between 10 and 15 min after chitin treatments and returned to basal level after 50 min (Figure 1B). Taken together, our data indicate that chitin induces ROS burst in barley leaves, but not in wheat leaves.

### Chitin-Induced ROS in Wheat Rachises and Rachis Nodes

**Figure 2:**
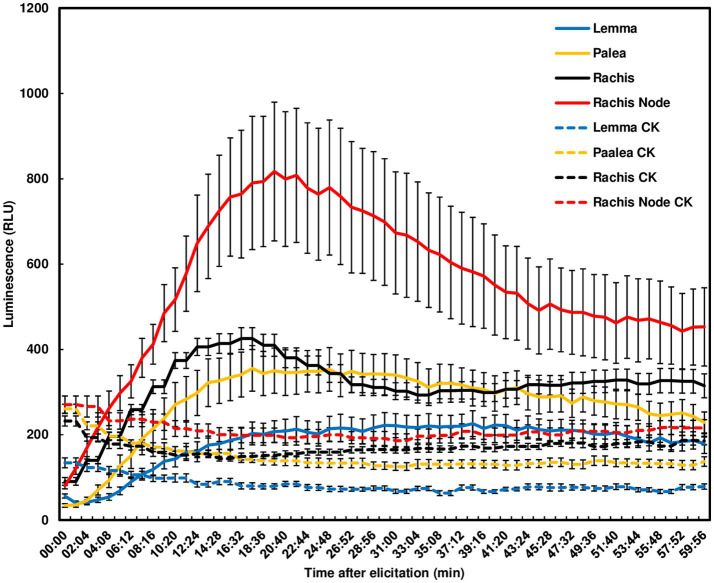
Chitin-triggered ROS in wheat head tissues. Spikes from the wheat variety, Norm, at mid-anthesis were dissected. Lemmas, paleae, rachises, and rachis nodes were used for ROS assays. Chitin (100 μg/ml) was used for treatment. The wells omitting chitin served as controls (CK). ROS were monitored using a chemiluminescence assay with L012 as a substrate. Signals (RLU) were recorded for 60 min after treatment. The data represent means ± standard error (*n* = 12) for each variety. The experiments were repeated three times with similar results.

Since *F. graminearum* primarily infects wheat head tissues and causes FHB, we examined chitin-triggered ROS in wheat head tissues. Heads from the FHB susceptible wheat variety Norm were dissected into paleae, lemmas, rachis nodes, and rachises for ROS assays. No ROS peak was detected in lemmas. A ROS peak reaching 800 relative light unit (RLU) was induced in rachis nodes at 20 min, slowly declined and did not return to basal levels at 60 min. Smaller ROS peaks were induced in rachises and paleae at 15 min (Figure 2). These observations demonstrate that chitin can trigger a relatively higher ROS burst in wheat rachis nodes than other wheat head tissues.

### Correlations Between DON, FHB Severity, and ROS Responses

**Figure 3:**
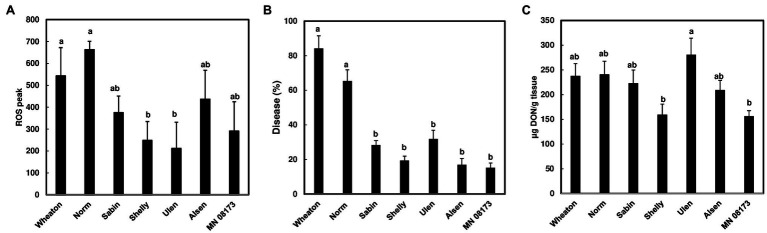
ROS level, FHB severity, and DON content in wheat varieties. **(A)** ROS peak in wheat rachis nodes. ROS were monitored using a chemiluminescence assay with L012 as a substrate. Signals (RLU) were recorded for 60 min after treatment. Chitin (100 μg/ml) was used. The data represent means ± standard error (*n* = 36) of the maximum luminescence for each variety; **(B)** FHB severity. Point inoculations (10 μl of 10^5^ conidia/ml) were performed on wheat florets with *F. graminearum* strain PH-1. FHB was scored as the percentage of spikelets infected at 21 days post-inoculation (dpi). Bars represent the average percentages and standard error of infected spikelets at 21 dpi for each variety. The mean for each variety was analyzed independently and compared by one-way ANOVA and Tukey’s honestly significant difference (HSD) *post-hoc* test using JMP15 (*n* = 10–20; *p* < .05); **(C)** DON content. DON was extracted from one or two infected spikelets from each head at 21 dpi and analyzed by GC/MS. The mean of DON for each variety was calculated and compared by one-way ANOVA and Tukey’s HSD *post-hoc* test using JMP15 (*n* = 10–20; *p* < .05). Different letters indicate significant difference.

**Figure 4:**
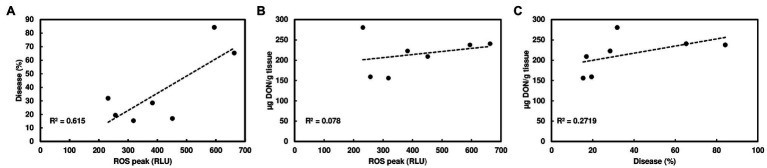
Correlation analyses of ROS, FHB, and DON among different wheat varieties. **(A)** A positive correlation between chitin-triggered ROS in wheat rachis nodes and FHB severity; **(B)** No correlation between chitin-triggered ROS and DON content; and **(C)** No correlation between FHB severity and DON content. The FHB score was measured as the percentage of spikelets with bleach or necrosis symptoms at 21 dpi. DON was extracted from one to two infected spikelets and analyzed by GC/MS at 21 dpi. ROS were monitored using a chemiluminescence assay with L012 as a substrate. Chitin (100 μg/ml) was used. ROS peaks (RLU) represent means ± standard error (*n* = 36) from each variety.

Since wheat rachis nodes have a ROS response, and the *F. graminearum* mutants deficient in DON production are unable to pass the rachis node, we assessed whether chitin-triggered ROS levels in rachis nodes were correlated with DON content and FHB severity. We examined ROS induction in rachis nodes from seven wheat varieties including FHB moderately resistant and susceptible varieties. We detected varied ROS induction in rachis nodes between different wheat varieties (Figure 3A). FHB severity was evaluated in these wheat varieties at 21 days after point inoculations. Two susceptible varieties, Norm and Wheaton, displayed higher disease levels compared to the other varieties (Figure 3B, Supplementary Figure S3). DON from infected florets varied from different varieties (Figure 3C). Our analyses showed relatively higher ROS levels in rachis nodes from FHB susceptible varieties, and a positive correlation between ROS levels and FHB severity from tested varieties (*R*^2^ = .615, *p* = .0368; Figure 4A). ROS levels were not correlated with DON content (*R*^2^ = .078, *p* = .5441; Figure 4B), and no correlation (*R*^2^ = .2719; *p* = .2300) was observed between FHB severity and DON content (Figure 4C). We also examined whether DON could interfere with ROS production in rachis nodes. Our assays showed that treating rachis nodes with DON did not affect ROS production induced by chitin (Supplementary Figure S4). Taken together, our results suggest that higher ROS in rachis nodes may promote FHB spread but not DON accumulation.

### Induction of Defense Genes in Wheat Heads and Rachis Nodes Treated With Chitin

**Figure 5:**
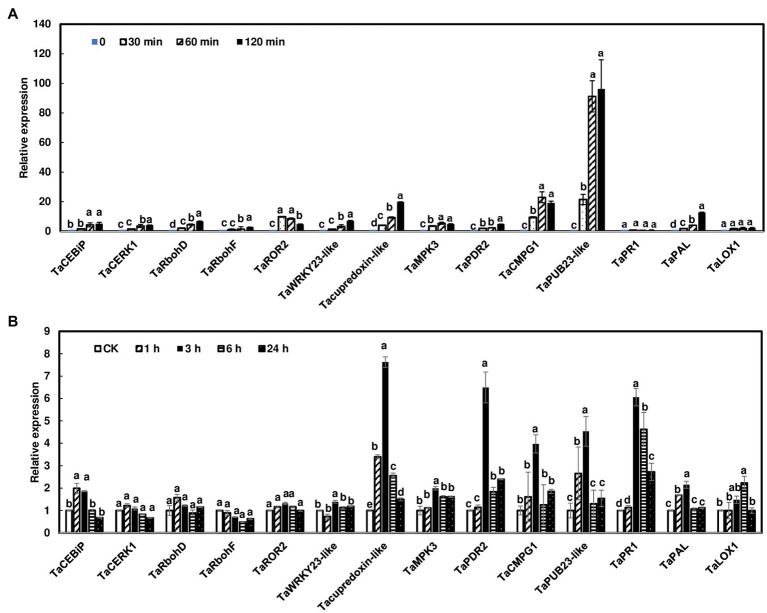
Induction of defense marker genes in wheat heads and rachis nodes treated with chitin. **(A)** Rachis nodes from Norm were dissected from flowering heads and placed in water overnight to avoid wound responses. The rachis nodes were treated with 100 μg/ml chitin for 0, 30, 60, and 120 min. Each treatment contained about 80 rachis nodes. **(B)** Wheat heads on live plants from Norm were dipped into .02% Tween with 100 μg/ml chitin or without chitin as controls. Heads were collected at 1, 3, 6, and 24 h. Each treatment contained three heads. The relative quantity of genes was determined using reverse transcriptase real-time PCR (RT-PCR). Wheat gene glyceraldehyde-3-phosphate dehydrogenase (*TaGAPDH*) was used as an internal control for transcript normalization. Gene induction fold was calculated relative to a mock control. The mean of expression for each gene was calculated and compared by one-way ANOVA and Tukey’s HSD *post-hoc* test using JMP15 (*n* = 3; *p* < .05). Different letters indicate significant difference.

A previous study showed that several wheat PTI marker genes were induced by chitin in wheat leaves (Schoonbeek et al., 2015). In addition, studies showed that salicylic acid (SA) and jasmonic acid (JA) play a sequential role in wheat defense against *F. graminearum* (Makandar et al., 2012; Ameye et al., 2015). Therefore, we assessed whether the transcript levels of a set of known defense genes were activated in wheat heads and rachis nodes treated with chitin. Since rachis node treatments and dissections from live plants would complicate the gene expression study, wheat rachis nodes were dissected from flowering heads and treated with chitin for 0, 30, 60, and 120 min. The PTI marker genes analyzed were activated in wheat rachis nodes in response to chitin treatments (Figure 5A). Most of the genes, such as *TaCERK1* and *TaCEBiP*, only showed a modest (<20-fold) increase in expression. In contrast, the ubiquitin ligase gene, *TaPUB23-like*, was induced approximately 100-fold with chitin treatment. In addition, most of these genes displayed the highest induction at 120 min after chitin treatment. The only exception was syntaxin *TaROR2*, which had the highest induction at 30 min, then decreased at 60 and 120 min (Figure 5A). Among hormone associated defense genes, the expression of *TaPAL1* was induced 10-fold at 120 min after chitin treatment. No induction of *TaPR*1 or *TaLOX1* was observed.

Furthermore, we assessed gene expression in wheat heads on live plants after chitin treatments. Since we observed that most defense genes were highly induced at 120 min in rachis nodes, we collected heads at 1, 3, 6, and 24 h after chitin treatment. Our data showed that most defense gene expressions peaked at 3 h and returned to normal expression at 24 h (Figure 5B). In addition, these gene inductions were relatively low compared to those in rachis nodes (Figure 5A). Taken together, these data suggest that chitin treatments can induce defense genes in wheat head tissues and detached rachis nodes.

### Expression of Defense Genes in Wheat Heads During *F. graminearum* Infection

**Figure 6:**
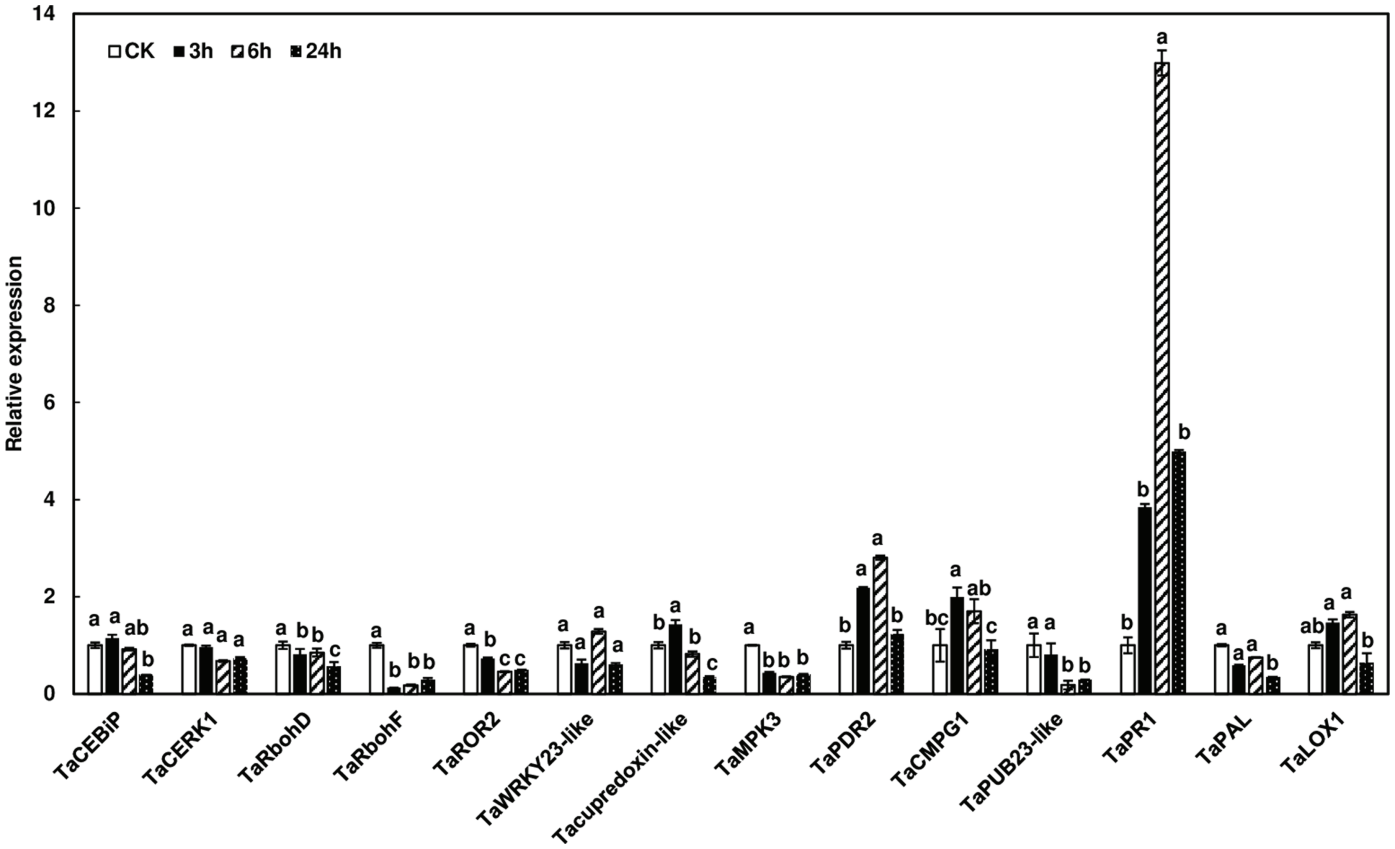
Induction of defense marker genes in wheat heads in response to *F. graminearum* infection. Wheat heads from Norm were dip-inoculated with PH-1 at a concentration of 10^5^ conidia/ml in .02% Tween 20. Wheat heads were collected at 0, 3, 6, and 24 hpi. Gene expression was determined by RT-qPCR. Wheat gene glyceraldehyde-3-phosphate dehydrogenase (*TaGAPDH*) was used as an internal control for transcript normalization. Fold changes of gene expression were relative to control samples. The mean of expression for each gene was calculated and compared by one-way ANOVA and Tukey’s HSD *post-hoc* test using JMP15 (*n* = 3; *p* < .05). Different letters indicate significant difference.

To assess defense-related gene expression during *F. graminearum* infection, we examined the expression of defense marker genes in wheat heads after *F. graminearum* inoculation. Since no wheat variety with type I resistance has been identified and gene expression was studied at initial infection stages, Norm wheat heads were collected at 0, 3, 6, and 24 h after whole head dip inoculation. Our results showed that some of the defense marker genes, such as *TaRbohF, TaMPK3*, and *TaPUB23-like* were not induced, while others, including *TaPR1* and *TaPDR2*, were induced during *F. graminearum* infection (Figure 6). Interestingly, *TaPUB23-like* that was highly induced (96-fold) in rachis nodes treated with chitin, was not induced during *F. graminearum* infection. On the other hand, *TaPR1* was moderately induced in chitin-treated wheat heads (6-fold) and *F. graminearum* infected heads (12-fold), but not in chitin-treated rachis nodes. Taken together, these results showed different defense gene expression patterns in rachis nodes treated with chitin, wheat heads treated with chitin, and wheat heads during *F. graminearum* infection.

## Discussion

Chitin-induced defense responses and signaling pathways are well studied in model plants, such as Arabidopsis and rice. However, limited studies are available in Triticeae, including wheat (Boyd et al., 2013). Our study provides novel information on chitin-trigged ROS in a tissue- and species-specific manner and indicates that ROS may play an important role during *F. graminearum* infection.

When sensing the presence of microbes, plants typically produce a rapid ROS burst, one of the earliest events during plant-pathogen interactions. Although we detected no ROS burst in wheat leaves from all tested varieties, we observed ROS responses in wheat head tissues, such as rachis nodes, induced by chitin (Figures 1, 2). A previous study attempted to measure the ROS burst in response to flg22 or chitin in wheat leaves but did not obtain reproducible results (Schoonbeek et al., 2015). Taken together, our and prior studies indicate no ROS burst induced by two well-studied PAMPs in wheat leaves. To the best of our knowledge, this is the first study showing that ROS responses were induced in wheat rachises and rachis nodes by chitin. These observations indicate a tissue-specific ROS response to chitin in wheat. It will be interesting to determine whether ROS responses to other PAMPs are tissue specific in wheat or other plant species. Although there is no ROS burst, defense marker genes are activated in wheat leaves after chitin treatment (Schoonbeek et al., 2015). In agreement with prior studies, we showed that defense genes were activated in wheat head tissues and rachis nodes treated with chitin. In Arabidopsis, after sensing the presence of elicitors, the earliest response is Ca^2+^ influx from the apoplast (Segonzac et al., 2011). Following the Ca^2+^ influx, two different branches of signaling occur as: one branch leads to ROS bursts, and the other leads to mitogen-activated protein kinases and associated defense gene upregulation (Segonzac et al., 2011). Collectively, these results suggest that only the defense gene pathway is activated in wheat leaves, but both ROS and defense gene pathways are activated in head tissues with chitin treatment. Alternatively, it is possible that there is increased ROS-scavenging activity in wheat leaves, and therefore, a ROS burst is not detected. Various ROS-scavenging enzymes, such as ascorbate peroxidases, glutathione, superoxide dismutases, and catalases, are important for ROS homeostasis in different plant tissues (Scandalios, 2005). Further investigations are needed to determine why different responses were observed from wheat leaves and head tissues, as well as whether ROS burst is affected by sampling the leaf disks from different development stages. Regarding ROS responses in barley leaves, we observed typical ROS bursts in all six varieties tested. Prior studies found a typical ROS response in barely leaf disks treated with chitin (Huckelhoven and Seidl, 2016). It will be interesting to determine why ROS responses are present in barley leaves but absent in wheat leaves.

We showed that chitin-triggered ROS in wheat rachises and rachis nodes were relatively low compared to barley and other plant species. For example, we observed that chitin induced higher ROS in barley leaves from some varieties, such as Golf. Chitin-induced high ROS responses have been detected in leaf disks of multiple dicotyledonous plants, such as *Arabidopsis* (Bredow et al., 2019) and *Nicotiana benthamiana* (Gimenez-Ibanez et al., 2018; Hao et al., 2019). For most monocots, luminol-based leaf disk assays are not effective (Melcher and Moerschbacher, 2016). Chitin-induced ROS bursts have been measured using cell suspension cultures in rice (Yamaguchi and Kawasaki, 2017) and wheat (Ortmann et al., 2004). In general, chitin induces a relatively weaker and less transient ROS production compared to flg22 (Melcher and Moerschbacher, 2016). For example, *Arabidopsis* leaf disks treated with chitin showed a lower ROS peak compared to leaf disks treated with flg22 (Melcher and Moerschbacher, 2016). Similarly, we observed a lower ROS peak triggered by chitin than flg22 in leaf disks from *N. benthamiana* (Hao et al., 2019). Typically, after elicitor treatment, a rapid and robust ROS burst peaks at approximately 10–20 min then returns to a basal level. In wheat rachis nodes, ROS displayed a slow and broad peak and did not return to a basal level at 60 min after chitin elicitation. Rachises and rachis nodes are critical for FHB spread throughout the wheat head. Therefore, we speculate that ROS may be involved in FHB spread. Our analyses demonstrated that ROS were induced by chitin in rachis nodes from FHB susceptible and moderately resistant wheat varieties. We observed relatively high ROS in FHB susceptible varieties and a potential correlation between ROS production in wheat rachis nodes and FHB spread (Figure 4). Since we only tested seven varieties, further investigations are needed to determine whether chitin-triggered ROS could serve as a simple selection marker for FHB resistance screening.

In general, ROS and its associated programmed cell death restrict biotrophic pathogen spread but promote necrotrophic pathogen growth. When colonizing rachis nodes, *F. graminearum* transitions to the necrotrophic stage. Therefore, increased ROS in rachis nodes may result in more cell damage and promote FHB spread. Prior studies showed oxidative stresses, such as fungicides at sublethal levels, induce H_2_O_2_ and facilitate DON production (Audenaert et al., 2010). H_2_O_2_, a key component of ROS, is suggested to be a signal for *F. graminearum* to produce DON, which in turn leads to increased H_2_O_2_ and DON production and promotes *F. graminearum* to further colonize the host plant (Desmond et al., 2008). However, we did not observe any correlation between ROS level and DON content from the tested wheat varieties. In addition, wheat rachis nodes treated with DON did not affect chitin-triggered ROS production (Supplementary Figure S2). Recently, a whole-plant live imaging platform has been applied to study local and systemic ROS signals in mature plants (Fichman et al., 2019). It is our interest to investigate ROS production and signaling using whole wheat live imaging during chitin treatment and *F. graminearum* infection.

We showed that both ROS and defense genes were activated in wheat rachis nodes treated with chitin. Transient cytosolic Ca^2+^ influx is the very first step that occurs in response to PAMP stimulation in Arabidopsis (Segonzac et al., 2011). However, little is known about Ca^2+^ signaling in wheat during PTI. Studies showed that calcium-dependent protein kinases are regulated in response to fungal infection in Triticeae (Huckelhoven and Seidl, 2016). Recently, one of the candidates for FHB resistance Fhb1, a quantitative trait locus discovered in Chinese germplasm Sumai 3, was identified as a histidine-rich calcium-binding protein that was predicted to interact with the antiporter CXIP4 that participates in modulation of the Ca^2+^ signaling (Li et al., 2019; Su et al., 2019). It is possible that the Ca^2+^ level is fine-tuned during FHB resistance between chitin-triggered apoplast Ca^2+^ influx and vacuole Ca^2+^ regulated by *TaHRC*. Further investigations are needed to elucidate the roles of Ca^2+^ signaling during *F. graminearum*-wheat interactions.

The downstream signaling genes are rapidly activated responding to elicitor treatment and pathogen infection. In Arabidopsis, after chitin treatment, over 180 genes were induced at 15 and 30 min and reached maximum induction at 30 or 60 min (Libault et al., 2007). However, we observed a weak or nonexistent induction for *TaCERK1*, *TaCEBiP*, *TaRbohD*, and *TaRbohF* in wheat heads and rachis nodes after chitin treatment. A few PTI marker genes were induced but reached their peak as late as 3 h after treatment (Figure 5). It is worth noting that *CMPG1* is one of the fastest genes induced with elicitor treatment. In parsley, it is activated within 5 min (Kirsch et al., 2001). In our study, *TaCMPG1* was slowly induced in chitin-treated wheat rachis nodes (induced at 30 min and peaked at 60 min) and heads (induced at 1 h and peaked at 3 h). Among the induced genes, the expression of *TaPub23-like* was highly induced in chitin-treated rachis nodes. The homologs of *TaPub23-like* are negative regulators for PTI in Arabidopsis (Trujillo et al., 2008), and therefore, its high induction in wheat rachis nodes may contribute to FHB spread. In addition, we detected different induction patterns of defense marker genes in chitin-treated wheat tissues and *F. graminearum* infected heads. The expression of *TaPUB23-like* was highly induced by chitin in wheat rachis nodes (96-fold), and 4-fold in chitin-treated heads, but was not induced in *F. graminearum* infected heads. *TaPAL*, which is involved in salicylate biosynthesis, showed a similar expression pattern as *TaPUB23-like* (Figures 5, 6). *F. graminearum* infection has a short biotrophic stage with extracellular hyphae advancing between live host cells without visible disease symptoms. This study suggests that wheat defense responses are relatively low during the *F. graminearum* biotrophic phase, which promotes fungal growth and colonization. One of the primary roles of effectors is to suppress PTI and ensure successful infection. Recently, our and other studies showed that several *F. graminearum* effectors suppress plant defense responses (Hao et al., 2019, 2020; Jiang et al., 2020). Further investigations are needed to compare these defense gene expressions in the wheat heads infected with the wild-type strain and effector mutants.

## Conclusion

We demonstrated that chitin-triggered ROS exhibited a tissue and species-dependent response in wheat and barley. We detected ROS burst in wheat rachis nodes from FHB susceptible and moderately resistant varieties. We showed a different induction pattern for defense genes in wheat tissues treated with chitin and infected with *F. graminearum*.

## Data Availability Statement

The original contributions presented in the study are included in the article/Supplementary Material, and further inquiries can be directed to the corresponding author.

## Author Contributions

GH conceived and designed the experiments and wrote the manuscript. HT, GH, and SM performed the experiment. GH and HT analyzed the data. All authors contributed to the article and approved the submitted version.

## Funding

This project was partially supported by US Department of Agriculture US Wheat and Barley Scab Initiative Grant No. FY20-HA-021. This work was also supported in part by the US Department of Agriculture, Agricultural Research Service.

## Conflict of Interest

The authors declare that the research was conducted in the absence of any commercial or financial relationships that could be construed as a potential conflict of interest.

## Publisher’s Note

All claims expressed in this article are solely those of the authors and do not necessarily represent those of their affiliated organizations, or those of the publisher, the editors and the reviewers. Any product that may be evaluated in this article, or claim that may be made by its manufacturer, is not guaranteed or endorsed by the publisher.

## Supplementary Material

Supplementary Figure S1 Dissection of wheat head tissues for ROS assays. Spikelets were removed from the middle of the flowering wheat head. The rachis node was dissected by cutting on both sides of the junction between the rachis and spikelet. Lemma and palea were gently separated using tweezers. Using a razor blade, the awn of the lemma was removed, and the lemma was cut into four quadrants. The palea was also cut, either into two or four pieces depending on its size. Rachises were cut between nodes and used for assays.

Supplementary Figure S2 No ROS burst detected in wheat leaves treated with various chitin concentrations. Wheat cultivar Norm leaves were treated with chitin concentrations of 100, 500, 1,000, and 2,000 μg/ml, respectively. Leaves were collected from 7- to 12-day old plants. Barley cultivar Golden Promise leaves treated with chitin (100 μg/ml) served as a positive control. Wheat leaves without chitin served as a negative control. ROS were monitored using a chemiluminescence assay with L012 as a substrate. The plates were run on a 96-well plate reader and signals (RLU) were recorded for about 40 min. The data represent means ± standard error (*n* = 12). The experiments were repeated twice with similar results.

Supplementary Figure S3 FHB symptoms in wheat cultivars inoculated with *F. graminearum* strain. Point inoculation (10 μl spore suspension containing 1,000 conidia) was performed on wheat florets with *F. graminearum* wild-type PH-1. Photographs were taken 21 days post-inoculation (dpi).

Supplementary Figure S4 No effect on ROS burst in rachis nodes treated with DON. Rachis nodes were removed from flowering heads and treated with 50 or 100 μg/ml DON overnight. The DON solution was replaced with ROS assay solution. Chitin (100 μg/ml) was used in the assay. ROS were monitored using a chemiluminescence assay with L012 as a substrate. Signals (RLU) were recorded for 60 min after treatment. The data represent means ± standard error (*n* = 12) for each treatment. The experiments were repeated twice with similar results.
