# Supplementary figures and images for: Chitin Triggers Tissue-Specific Immunity in Wheat Associated With Fusarium Head Blight
Source: Front Plant Sci. 2022 Feb 9;13:832502. doi: 10.3389/fpls.2022.832502 (PMC8864176; doi:10.3389/fpls.2022.832502)

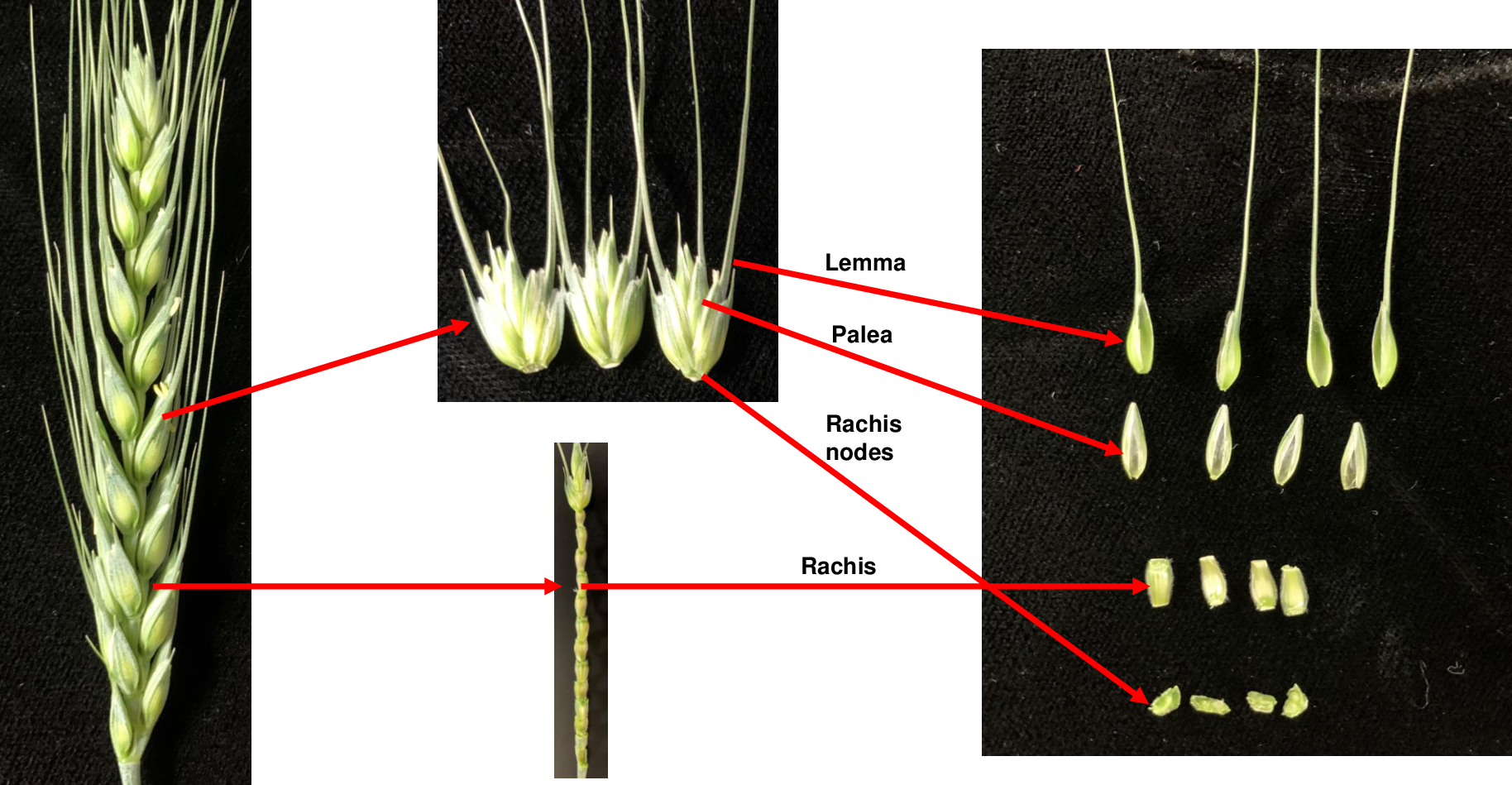

Supplement: Supplementary Figure S1 — Dissection of wheat head tissues for ROS assays. Spikelets were removed from the middle of the flowering wheat head. The rachis node was dissected by cutting on both sides of the junction between the rachis and spikelet. Lemma and palea were gently separated using tweezers. Using a razor blade, the awn of the lemma was removed, and the lemma was cut into four quadrants. The palea was also cut, either into two or four pieces depending on its size. Rachises were cut between nodes and used for assays. [file Image_1.TIF]

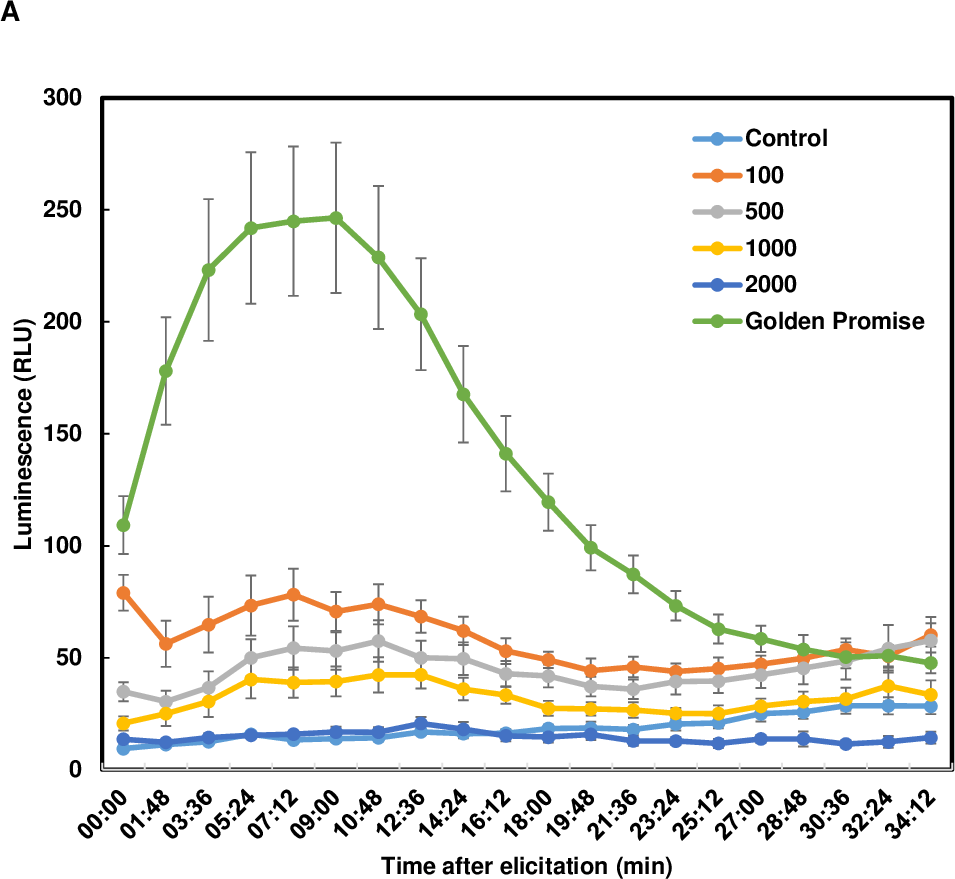

Supplement: Supplementary Figure S2 — No ROS burst detected in wheat leaves treated with various chitin concentrations. Wheat cultivar Norm leaves were treated with chitin concentrations of 100, 500, 1,000, and 2,000 μg/ml, respectively. Leaves were collected from 7- to 12-day old plants. Barley cultivar Golden Promise leaves treated with chitin (100 μg/ml) served as a positive control. Wheat leaves without chitin served as a negative control. ROS were monitored using a chemiluminescence assay with L012 as a substrate. The plates were run on a 96-well plate reader and signals (RLU) were recorded for about 40 min. The data represent means ± standard error (n = 12). The experiments were repeated twice with similar results. [file Image_2.TIF]

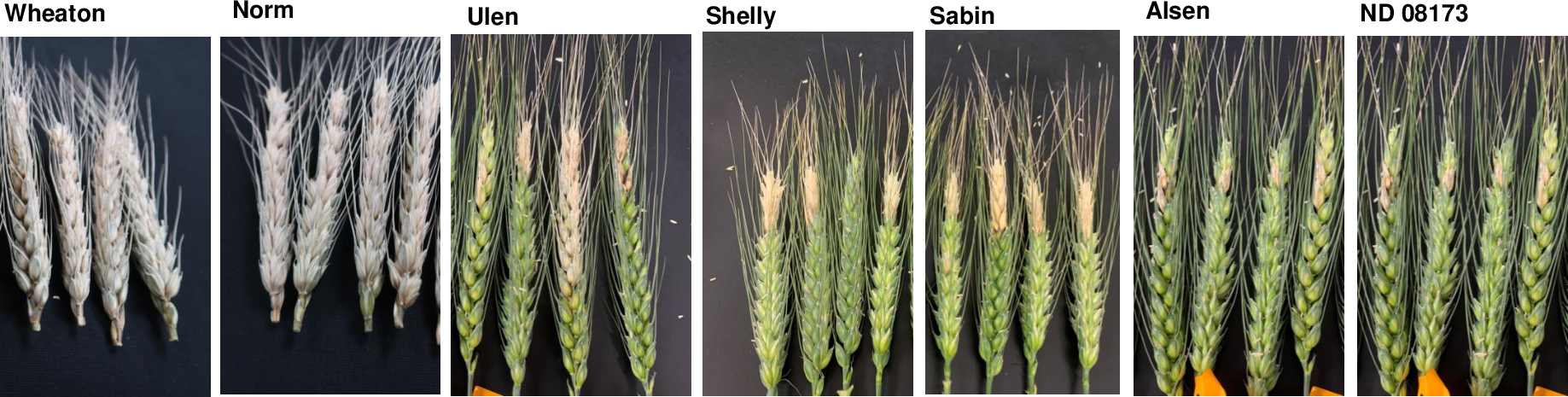

Supplement: Supplementary Figure S3 — FHB symptoms in wheat cultivars inoculated with F. graminearum strain. Point inoculation (10 μl spore suspension containing 1,000 conidia) was performed on wheat florets with F. graminearum wild-type PH-1. Photographs were taken 21 days post-inoculation (dpi). [file Image_3.TIF]

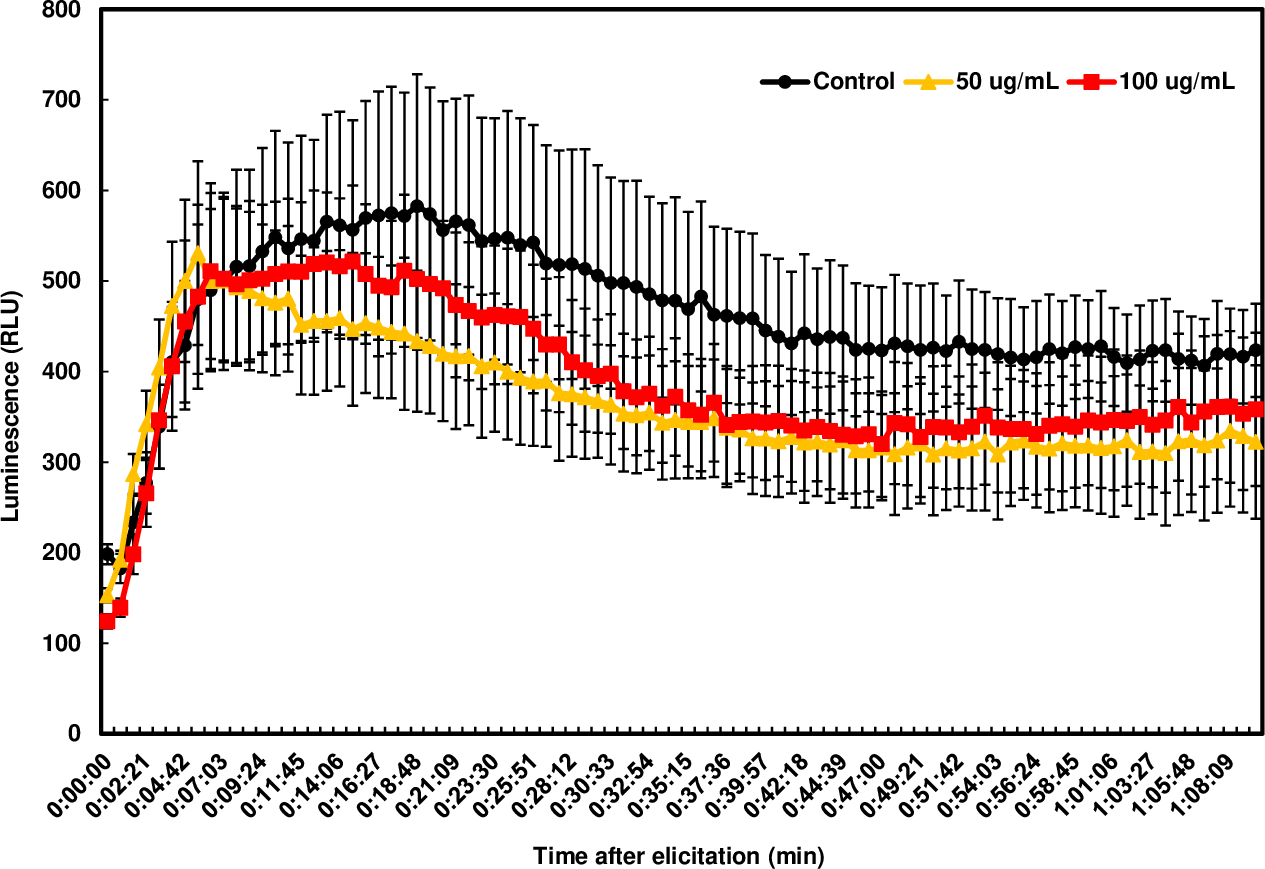

Supplement: Supplementary Figure S4 — No effect on ROS burst in rachis nodes treated with DON. Rachis nodes were removed from flowering heads and treated with 50 or 100 μg/ml DON overnight. The DON solution was replaced with ROS assay solution. Chitin (100 μg/ml) was used in the assay. ROS were monitored using a chemiluminescence assay with L012 as a substrate. Signals (RLU) were recorded for 60 min after treatment. The data represent means ± standard error (n = 12) for each treatment. The experiments were repeated twice with similar results. [file Image_4.TIF]
