# Supplementary material for: Chitin Triggers Tissue-Specific Immunity in Wheat Associated With Fusarium Head Blight
Source: Front Plant Sci. 2022 Feb 9;13:832502. doi: 10.3389/fpls.2022.832502 (PMC8864176; doi:10.3389/fpls.2022.832502)
Supplement: Supplementary file 5 [file Table_1.docx]

**Table S1.** Primers used in this study

|  |  |  |  |  |
| --- | --- | --- | --- | --- |
| Primer | Sequence | Reference |  |  |
|  |  |  |  |  |
| TaMPK3-RT-F | 5'- ACCCTTACCTAGAGCGGCTTC -3' | Schoonbeek et al., 2015 |  |  |
| TaMPK3-RT-R | 5'- ACTCCAGGGCTTCGTTGAATA -3' |  |  |  |
|  |  |  |  |  |
| TaRor2-RT-F | 5'- TCGTGCTCAAGAACACCAAC -3' | Schoonbeek et al., 2015 |  |  |
| TaRor2-RT-R | 5'- AATCGAGTGGCTCAACGAAC -3' |  |  |  |
|  |  |  |  |  |
| TaWRKY23-RT-F | 5'- GAGCGTAGACGTCAGCACCA -3' | Schoonbeek et al., 2015 |  |  |
| TaWRKY23-RT-R | 5'- CACGGATGCTAATGGCCACC -3' |  |  |  |
|  |  |  |  |  |
| TaPDR2-RT-F | 5'- GTGCAGGGGATTCAGTACACA -3' | Schoonbeek et al., 2015 |  |  |
| TaPDR2-RT-R | 5' -GTATGTTTGCGAGCATGGAAG -3' |  |  |  |
|  |  |  |  |  |
| TaPUB23-like-RT-F | 5'- CGTTCATCAGAATGCTCAGCTG -3' | Schoonbeek et al., 2015 |  |  |
| TaPUB23-like-RT-R | 5'- TTCTCTTTTGTAGGCACGAACCA -3' |  |  |  |
|  |  |  |  |  |
| TaCMPG1-like-RT-F | 5'- GGACGCAACCAAGGAGAAGA -3' | Schoonbeek et al., 2015 |  |  |
| TaCMPG1-like-RT-R | 5'- TTGAGCCCTCTGAAGTCCAT -3' |  |  |  |
|  |  |  |  |  |
| TaCupredoxin-RT-F | 5'- AGCGGTAACCTACAACGTCG -3' | Schoonbeek et al., 2015 |  |  |
| TaCupredoxin-RT-R | 5'- GACGATGTCATCACCCACGT -3' |  |  |  |
|  |  |  |  |  |
| TaCEBiP-RT-F | 5'- CAGCAACACCTCGGATGAT -3' | This study |  |  |
| TaCEBiP-RT-R | 5'- GCACTGCGGTGCAATTTAG -3' |  |  |  |
|  |  |  |  |  |
| TaRbohD-RT-F | 5'- ACCACCAGACCAGACCAGAC -3' | Dmochowska-Boguta et al., 2013. |  |  |
| TaRbohD-RT-R | 5'- TGGTTGGATAGGAGGCGTAG -3' |  |  |  |
|  |  |  |  |  |
| TaRbohF-RT-F | 5'- TGGCACCCCTTCTCAATTAC -3' | Dmochowska-Boguta et al., 2013. |  |  |
| TaRbohF-RT-R | 5'- CTCTCGTGTCCAGTCACCAA -3' |  |  |  |
|  |  |  |  |  |
| TaCERK1-RT-F | 5'- TTACCCCATCGACGCCATTC -3' | Lee et al., 2014 |  |  |
| TaCERK1-RT-R | 5'- TCTGCCGGACATGAGGTTCA -3' |  |  |  |
|  |  |  |  |  |
| TaPR1-RT-F | 5'- CGTCTTCATCACCTGCAACTA -3' | Ameye et al., 2015 |  |  |
| TaPR1-RT-R | 5'- CAAACATAAACACACGCACGTA -3' |  |  |  |
|  |  |  |  |  |
| TaPAL-RT-F | 5'- TTGATGAAGCCGAAGCAGGACC -3' | Ameye et al., 2015 |  |  |
| TaPAL-RT-R | 5'- ATGGGGGTGCCTTGGAAGTTGC -3' |  |  |  |
|  |  |  |  |  |
| TaLOX1-RT-F | 5'- GGCACGCCATCGAGCAGTACG -3' | Ameye et al., 2015 |  |  |
| TaLOX1-RT-R | 5'- TACTGCCCGAAGTTGACCGCC -3' |  |  |  |
|  |  |  |  |  |
| TaGAPDH-RT-F | 5'- TTGCTCTGAACGACCATTTC -3' | This study |  |  |
| TaGAPDH-RT-R | 5'- GACACCATCCACATTTATTCTTC -3' |  |  |  |
|  |  |  |  |  |
|  |  |  |  |  |
